# Factors Associated with the Need for Insulin as a Complementary Treatment to Metformin in Gestational Diabetes Mellitus

**DOI:** 10.1055/s-0039-1700796

**Published:** 2019-12

**Authors:** Matheus Leite Ramos de Souza, Rodrigo Ribeiro e Silva, Thiago Ribeiro e Silva, Larissa Cano de Oliveira, Guilherme Dienstmann, Iramar Baptistella do Nascimento, Jean Carl Silva

**Affiliations:** 1 Department of Medicine, Universidade da Região de Joinville, Joinville, SC, Brazil; 2 Department of Medicine, Universidade Positivo, Curitiba, PR, Brazil

**Keywords:** gestational diabetes, metformin, insulin, combined treatment, diabetes gestacional, metformina, insulina, tratamento combinado

## Abstract

**Objective** To evaluate the factors associated with the need for insulin as a complementary treatment to metformin in pregnant women with gestational diabetes mellitus (GDM).

**Methods** A case-control study was performed from April 2011 to February 2016 with pregnant women with GDM who needed complementary treatments besides diet and physical exercise. Those treated with metformin were compared with those who, in addition to metformin, also needed the combination with insulin. Maternal characteristics and glycemic control were evaluated. Multinomial logistic regression models were developed to evaluate the influence of different therapies on neonatal outcomes.

**Results** A total of 475 pregnant women who needed pharmacological therapy were evaluated. Of these, 366 (77.05%) were submitted to single therapy with metformin, and 109 (22.94%) needed insulin as a complementary treatment. In the analysis of the odds ratio (OR), fasting glucose (FG) < 90 mg/dL reduced the odds of needing the combination (OR: 0.438 [0.235–0.815]; *p* = 0.009], as well as primiparity (OR: 0.280 [0.111–0.704]; *p* = 0.007]. In obese pregnant women, an increased chance of needing the combination was observed (OR: 2,072 [1,063–4,039]; *p* = 0,032).

**Conclusion** Obesity resulted in an increased chance of the mother needing insulin as a complementary treatment to metformin, while FG < 90 mg/dL and primiparity were protective factors.

## Resumo

**Objetivo** Avaliar os fatores associados à necessidade de insulina como tratamento complementar à metformina em gestantes com diabetes mellitus gestacional (DMG).

**Métodos** Um estudo caso-controle foi realizado de abril de 2011 a fevereiro de 2016 com gestantes portadoras de DMG que necessitaram de tratamentos complementares além de dieta e exercícios físicos. Aquelas tratadas com metformina foram comparadas com aquelas que, além da metformina, também precisaram de combinação com insulina. Foram avaliadas as características maternas e de controle glicêmico. Modelos de regressão logística multinomial foram construídos para avaliar a influência das diferentes terapias nos desfechos neonatais.

**Resultados** Foram avaliadas 475 gestantes que necessitaram de terapia farmacológica. Destas, 366 (77,05%) utilizaram terapia única com metformina, e 109 (22,95%) necessitaram de insulina como tratamento complementar. Na análise da razão de possibilidades (RP), a glicemia de jejum (GJ) < 90 mg/dL reduziu as chances de necessidade da combinação (RP: 0,438 [0,235–0,815]; *p* = 0,009), bem como a primiparidade (RP: 0,280 [0,111–0,704]; *p* = 0,007). Em gestantes obesas, foi observada uma chance maior de necessidade da combinação (RP: 2.072 [1.063–4.039]; *p* = 0,032).

**Conclusão** A obesidade resultou em um aumento na chance de a mãe precisar de insulina como tratamento complementar à metformina, enquanto a GJ < 90 mg/dL e a primiparidade foram fatores de proteção.

## Introduction

Gestational diabetes mellitus (GDM) is a metabolic alteration with prevalence between 3% and 25%, depending on the ethnic group and the diagnostic criteria used.^1^ ^2^ In the last decades, there has been a progressive increase in the number of pregnant women diagnosed with diabetes as a result of population growth, increased maternal age, lack of physical activity, and an increased prevalence of obesity.^3^

The maternal hyperglycemia that is a characteristic of GDM has a negative impact on the progression of pregnancy.^4^ ^5^ Therefore, GDM is an independent risk factor for obstetric complications, such as: preterm delivery; preeclampsia; large for gestational age (LGA) newborns and macrossomics; birth traumas, such as dystocia; increased need for cesarean sections; and neonatal hypoglycaemia.^6^ ^7^

The initial treatment recommended is lifestyle changes, such as diet and physical activity.^8^ ^9^ When such measures are not sufficient to reach adequate glucose levels, pharmacological therapy is required, with metformin or insulin.^10^ It is believed that ∼ 15% to 60% of patients require pharmacological treatment in combination with diet and physical activity to achieve control of the condition.^11^

Metformin is an oral anti-hyperglycemic drug derived from biguanide that has its main site of action in the liver. The three main mechanisms of action are: reduction of hepatic gluconeogenesis, reduction of glucose absorption by the gastrointestinal tract, and improvement in the use of peripheral glucose by increasing cellular sensitivity to insulin.^12^ It was initially developed for use in type-2 diabetes mellitus (DM2) and, because it crosses the placental barrier, its administration in cases of GDM was delayed. It had its safety proven in pregnancy as it was used for fertility treatments in patients with polycystic ovary syndrome; these patients kept using the medication throughout pregnancy.^13^ ^14^ Thus, because it is a safe drug, more cost-effective and easier to use compared with insulin, it is indicated in cases of GDM because it is metabolically similar to DM2.^15^

However, even with metformin being as effective as insulin in glycemic control, in some patient profiles it is significantly associated with a low response to the monotherapy and, therefore, needs to be supplemented with insulin.^16^

Given this context, the objective of the present study was to evaluate maternal and glycemic control factors that influence the chance of pregnant women with GDM needing insulin as a complementary treatment to metformin.

## Methods

A case-control study was conducted. The sample was composed of pregnant women with GDM, and those who needed only treatment with metformin were compared with pregnant women treated with metformin who needed to be associated with insulin.

Sample size was defined for convenience, covering all pregnant women who met the inclusion criteria. The women were cared for in the period from April 2011 to February 2016 at the High-Risk Care Service of Maternidade Darcy Vargas (MDV), in the city of Joinville, state of Santa Catarina, Brazil. All of the patients had their deliveries performed at the same service.

The inclusion criteria were: pregnant women older than 18 years of age, with a diagnosis of GDM, need for pharmacological therapy complementary to diet and physical exercise, and complete data in the electronic patient record (EPR). The exclusion criterion was participants with incomplete data in the EPR. However, there was no need for exclusions throughout the study.

The project was approved under CPAE number 82477318.1.0000.5363 by the Research Ethics Committee (REC) of Hospital Regional Hans Dieter Schmidt , in the city of Joinville. We also followed item 32 of the Declaration of Helsinki, which states that in cases in which consent is impossible or impracticable to obtain, research may be done only after consideration and approval of a research ethics committee. Thus, the present study only started after the REC's approval opinion, and followed in its development the requirements of Resolution 466/12 of the Brazilian National Health Council of the Ministry of Health.

The pregnant women included had been diagnosed at the MDV service when they were being followed up according to the guidelines of the Brazilian Diabetes Society (Sociedade Brasileira de Diabetes SBD, in Portuguese), which are the same as those of the World Health Organization (WHO). Screening for gestational age (GA) below 20 weeks was performed by examining fasting glucose (FG). The diagnosis of GDM was established when the result was between 92 mg/dL and 125 mg/dL. From the 24th week of gestation, all of the patients were submitted to the oral glucose tolerance test (OGTT). The reference values for GDM are: FG ≥ 92 mg/dL, glycemia after 1h ≥ 180 mg/dL, or ≥ 153 mg/dL after 2h, and any one of the points being altered in the curve already defines the diagnosis.^1^

All of the patients analyzed had been submitted at the time to the same follow-up by the MDV multiprofessional team. As soon as they arrived at the hospital, they sat through lectures with nutritionists, physiotherapists and psychologists. After this, the same team continued the follow-up in an individualized way and then started the medical consultations.

The nutritional instructions were established individually and according to the maternal body mass index (BMI) on the day of the consultation, following models similar to those used for the general population. The importance of having a balanced diet was always emphasized. As recommended by the SBD, the pregnant women were advised that the food intake should be composed of 40% to 55% of carbohydrates, 20% to 35% of fat, and 15% to 20% of protein, and that 3 smaller meals (morning, afternoon and evening snacks) and 3 larger ones should be made.^1^

Physical activity, as a complementary treatment to the diet therapy, was recommended as follows: performing low-impact aerobic activities without risk of falls, such as walking, swimming and cycling. The intensity should be low or moderate, with a duration between 30 and 45 minutes and a frequency of 3 times a week.^17^

The routine that was used during each medical consultation to define the therapeutic proposal was based on a clinical-laboratory score, which consists of 5 parameters with scores ranging from -2 to +2. The criterias evaluated were: FG, postprandial blood glucose, fetal abdominal circunference, maternal BMI, and GA at the visit. Thus, four recommendations were made according to the total score of the factors added. Scores below 0 (zero) indicate the need for a new consultation with a nutritionist; between 0 and 2, maintenance of diet and exercise; between 2 and 4, introduction of oral antihyperglycemic; and, when greater than 4, administration of insulin added to the oral drug.^18^

All data were collected at the time of the study from the EPR. The maternal variables analyzed were: age, parity, pregestational BMI, weight gain during pregnancy, diagnostic gestational age (GA), preeclampsia, presence of systemic arterial hypertension (SAH) prior to gestation, 75-gram oral glucose tolerance test (OGTT), controlled glycemic index during prenatal care, glycosylated hemoglobin (HbA1c), and delivery route.

The data collected from the newborns were: GA at birth, prematurity, birth weight, Apgar score, need for admission to the neonatal intensive care unit (ICU), presence of malformations and deaths.

The collected data were analyzed using the Statistical Package for the Social Sciences (SPSS, IBM Corp., Armonk, NY, US) software, version 21. The quantitative data were processed through the calculation of means and standard deviations. Absolute and relative frequencies were calculated for the qualitative variables. To verify the hypothesis of equality between the means of the groups, the Student *t*-test was used when the distribution was normal, and the Mann-Whitney non-parametric test was used when the normality test was rejected. The normal test used was the Kolmogorov-Smirnov test. In order to test the homogeneity of the groups in relation to the proportions, the Chi-squared test or the Fisher exact test were used for frequencies lower than 5.

Multinomial logistic regression models were developed to analyze the influence of the gestational diabetes diagnosis on the studied outcomes. The significance of the effect of the variables was estimated by the odds ratio (OR), with the respective 95% confidence intervals (95%CI). Values of *p* < 0.05 were considered significant.

## Results

**Fig. 1:**
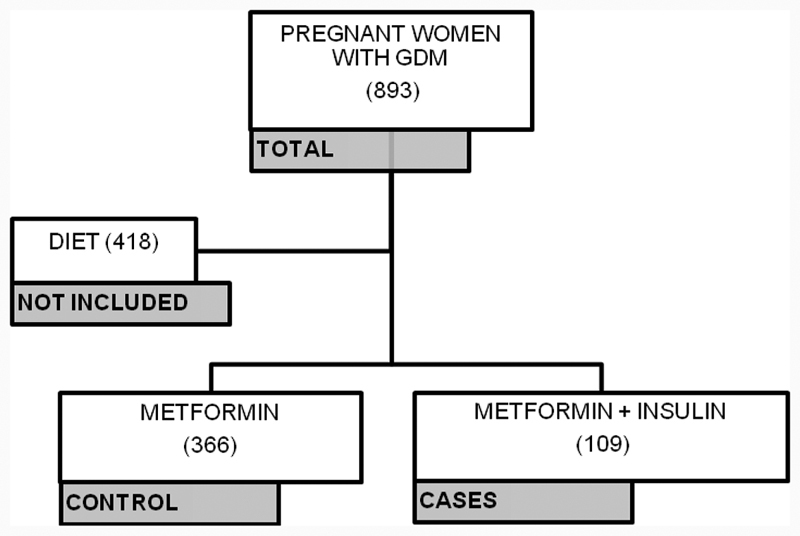
Flowchart of the inclusion of the participants in the study.

During the study period, 893 pregnant women with GDM were followed up. Of these, 418 underwent treatment only with diet and physical exercises and, therefore, were not included in the study. A total of 475 pregnant women were enrolled in the study, 366 (77.05%) of whom used only metformin as pharmacological therapy for GDM, and 109 (22.95%) needed insulin to complement the metformin. There were no exclusions of participants throughout the study (Fig. 1).

As shown in Table 1, some maternal characteristics were more related to failure in single metformin therapy. In the group that needed complementation, we observed: older maternal age, greater number of previous pregnancies, greater weight gain, and earlier diagnosis. We also observed that pregnant women with a higher BMI had a greater need for the metformin and insulin combination, especially obese women, who represented 55.04% of the combination group.

**Table 1:** Maternal characteristics

	Metformin (*n* = 366)	Combination (*n* = 109)	*p*-value
**Age**	31.48 (6.88)	34.40 (6.33)	0.020^c^
**Previous pregnancies**	3.00 (2.08)	3.50 (1.73)	0.008^c^
**BMI**	29.48 (5.96)	32.13 (6.99)	0.009^c^
**BMI class**			
* Low weight*	28 (7.65%)	1 (0.91%)	0.006^b^
* Adequate*	78 (21.31%)	17 (15.59%)	0.190^a^
* Overweight*	116 (31.69%)	29 (26.60%)	0.311^a^
* Obese*	139 (37.97%)	60 (55.04%)	0.002^a^
**Weight gain**	1.31 (3.16)	3.48 (3.72)	0.009^c^
**GA at diagnosis**	28.76 (5.83)	25.95 (5.76)	< 0.001^c^
**SHGS**	21 (5.73%)	9 (8.25%)	0.346^a^
**Previous SAH**	55 (15.02%)	17 (15.59%)	0.864^a^

Abbreviations: BMI, body mass index; GA, gestational age; SHGS, specific hypertensive gestation syndrome; SAH, systemic arterial hypertension.

Notes: ^a^Chi-squared test; ^b^Fisher exact test; ^c^Mann-Whitney test. Mean and standard deviation, absolute numbers and percentages.

We identified that the pregnant women with higher values of FG in the OGTT belonged to the group that represented the failure of the metformin monotherapy. Likewise, fasting and postprandial glycemic control data were higher in the group that required the combination. Furthermore, the third-trimester HbA1c had a higher percentage in this same group (Table 2).

**Table 2:** Characteristics related to diabetes

	Metformin (*n* = 366)	Combination (*n* = 109)	*p*-value
**OGTT**			
* Fasting*	92.77 (11.90)	98.30 (7.42)	< 0.001^c^
* 1 hour*	172.16 (33.90)	178.50 (32.58)	0.769^c^
* 2 hours*	147.33 (36.58)	151.35 (30.67)	0.403^c^
**Glycemic control**			
* Fasting*	88.90 (9.81)	98.29 (10.58)	<0.001^c^
* Postprandial*	120.28 (14.16)	124.03 (15.97)	<0.001^c^
* HBA1C*	5.35 (0.34)	5.60 (0.38)	0.018^c^

Abbreviations: HBA1C, A1c glycated hemoglobin; OGTT, oral glucose tolerance test.

Notes: ^c^Mann-Whitney test. Mean and standard deviation, absolute numbers and percentages.

Table 3 shows that there was a significant difference regarding birth weight between the newborns of the groups, which results in the finding of a higher percentage of large for gestational age newborns (LGA NB) composing the combination group, and a greater number of adequate for gestational age newborns (AGA NB) in the metformin group.

**Table 3:** Newborn characteristics

	Metformin (*n* = 366)	Combination (*n* = 109)	*p*-value
**GA at birth**	38.67 (1.36)	38.55 (1.57)	0.228^c^
**Premature**	17 (4.64%)	4 (3.66%)	0.664^b^
**Delivery route**			
* Normal*	174 (47.54%)	41 (37.61%)	0.068^a^
* Caesarean*	192 (52.46%)	68 (62.38%)	0.068^a^
**Weight**	3334.18 (357.52)	3461.25 (448.55)	0.001^c^
**Weight Classification**			
* SGA*	9 (2.45%)	1 (0.91%)	0.467^b^
* AGA*	292 (79.78%)	75 (68.80%)	0.016^a^
* LGA*	65 (17.75%)	33 (30.29%)	0.005^a^
**Apgar**			
* 1 minute*	8.03 (1.20)	7.75 (1.37)	0.227^c^
* 1 minute low*	28 (7.65%)	7 (6.42%)	0.806^a^
* 5 minutes*	9.01 (0.69)	8.75 (0.78)	0.477^c^
* 5 minutes low*	2 (0.54%)	2 (1.83%)	0.196^b^
**Need for ICU**	15 (4.09%)	15 (13.76%)	< 0.001^a^
**Malformation**	13 (3.55%)	2 (1.83%)	0.537^b^
**Death**	2 (0.54%)	0	1.000^b^

Abbreviations: AGA, adequate for gestational age; GA, gestational age; ICU, intensive care unit; LGA, large for gestational age; SGA, small for gestational age.

Notes: ^a^Chi-squared test; ^b^Fisher exact test; ^c^Mann-Whitney test. Mean and standard deviation, absolute numbers and percentages.

Finally, Table 4 shows that primiparity and FG < 90 mg/dL reduce the chance of failure of the single therapy with metformin, and, therefore, they represent protective factors. On the other hand, obesity was found to cause an increase in the chance of need for insulin supplementation. The other factors analyzed did not show a significant influence.

**Table 4:** Multinomial analysis of the factors associated with the need for complementary therapy to the insulin therapy

	MTF/MTF + insulin	OR^a^	95%CI	*p*-value
**Age > 30 years**	164/197	0.879	0.508–1.521	0.645
**Primiparity**	285/76	0.280	0.111–0.704	0.007
**GA diagnosis before the 28^th^ week**	213/148	1.331	0.654–2.708	0.430
**Low weight**	338/23	0.300	0.038–2.396	0.256
**Obese**	208/153	2.072	1.063–4.039	0.032
**FG < 90 mg/dL**	225/136	0.438	0.235–0.815	0.009

Abbreviations: 95%CI, 95% confidence interval; FG, fasting glucose; GA, gestational age; MTF, metformin; OR, odds ratio.

a Gross odds ratio.

## Discussion

The present study aimed to define the maternal and glycemic control factors that would be predictors of the need to use insulin as a complementary treatment to metformin among pregnant women with GDM. It was possible, therefore, to establish an OR for the main outcomes. Primiparity and FG < 90 mg/dL were deemed protective factors, and maternal obesity, a factor of increased chance.

In the composition of the groups, a 22.95% failure rate of the metformin monotherapy was found, which is similar to the results found in other studies.^11^ ^16^ Silva et al,^11^ for example, in a clinical trial comparing different hypoglycemic agents, observed a 21.2% rate of need for insulin supplementation in the group that used metformin. Ashoush et al^16^ observed a rate of 23.4%.

Although McGrath et al^19^ did not describe any differences, we observed in their article that the maternal age was different between the groups, and higher in those who needed the association (*p* = 0.02), as observed by other authors.^20^ ^21^ However, maternal age > 30 years did not increase the chance of metformin failure (OR: 0.879 [95%CI = 0.508–1.521]; *p* = 0.645), a result that was contrary to expectations. The study by Gante et al.^22^ for example, showed age as a significant risk factor (OR: 1.08 [95%CI = 1.03–1.13]; *p* = 0.003); Khin et al^23^ also observed this phenomenon. Neither of the studies, however, hypothesized this result.

Differently from maternal age, a significant association was found between the number of previous pregnancies and metformin failure. There was a difference between the groups (*p* = 0.008), and the effect measure analysis showed that women in the first gestation (primiparous women) are less likely to require supplementation with insulin. Ashoush et al^16^ did not find the same significant relationship; however, their sample was smaller than that of the present study.

Moreover, the group that required insulin supplementation had earlier diagnoses (*p* < 0.001), in the same way as the populations of other studies.^19^ ^22^ Therefore, it would be expected that those women who developed GDM earlier in pregnancy were those with higher risk factors and, therefore, the diagnostic GA was a predictor of metformin failure. However, no increase in odds was found in patients requiring pharmacotherapy with GA < 28 weeks (OR: 1.331 [95%CI = 0.654–2.709]; *p* = 0.430). In disagreement with the study by Khin et al,^23^ which was published recently, we report an OR of 1.12 (95%CI = 1.1–1.2).

Maternal pregestational BMI is a factor that a large part of the studies described as impacting the effectiveness of the single therapy with metformin. In the present study, a higher BMI was observed in the group that required insulin therapy, which had more pregnant women classified as obese than the other group. Therefore, an increased chance was obtained (OR: 2.072 [95%CI = 1.063–4.039]; *p* = 0.032) for the occurrence of such an event. According to two studies^22^ ^24^ that also obtained similar results (OR: 1.06 [95%CI = 1.02–1.10] and OR: 4.10 [95%CI = 1.46–11.51]), this is explained due to the fact that higher BMIs and obesity increase insulin resistance and decrease the sensitivity of the oral hypoglycemic.^22^ ^24^ Sales et al,^25^ proving the failure of the drug in this context, when evaluating its impact on the outcomes (reduction of BMI and prevention of GDM in obese pregnant women), observed that metformin was not effective.

Finally, FG, when < 90 mg/dL in the OGTT, is a protective factor for the need for insulin supplementation (Table 4). Therefore, pregnant women with these levels of FG have a good chance of responding to metformin monotherapy. Ashoush et al,^16^ Tertti et al^21^ and Gante et al^22^ also detected this characteristic. Similarly, Silva et al^15^ found a lower fasting blood glucose value related to the success of metformin in their study. Finally, still corroborating this finding, a study^19^ that also had the objective of evaluating the predictors of insulin supplementation, even though it did not find significance, stated that the predominant factor for such a need would be high FG.^19^

The present study had some limitations, such as the case-control design, which does not offer the highest level of evidence possible. Another bias present is information due to the fact that retrospective data present in the EPR were collected. The strengths of the study are: the large sample size and the diagnosis and follow-up of all patients in a single hospital. Therefore, we can state that the present study contributed to the improvement of the scientific knowledge regarding the identification of subgroups of patients who need more attention because they have a greater chance of needing a combination of insulin and metformin as treatment.

## Conclusion

In conclusion, despite the differences found among the populations, only obesity resulted in an increased chance that the pregnant woman needed insulin as a complementary treatment to metformin, while FG < 90 mg/dL and primiparity were protective factors.
